# Key components of early intervention programs for preterm infants and their parents: a systematic review and meta-analysis

**DOI:** 10.1186/1471-2393-13-S1-S10

**Published:** 2013-01-31

**Authors:** Karen M Benzies, Joyce E Magill-Evans, K Alix Hayden, Marilyn Ballantyne

**Affiliations:** 1 Faculty of Nursing, University of Calgary, 2500 University Drive NW, Calgary, AB T2N 1N4 Canada; 2 Department of Occupational Therapy, 2-64 Corbett Hall, University of Alberta, Edmonton, AB T6G 2G4 Canada; 3 Libraries and Cultural Resources, University of Calgary, 2500 University Drive NW, Calgary AB T2N 1N4 Canada; 4 School of Nursing, McMaster University, 1280 Main Street West, Hamilton, ON L8S 4K1 Canada

## Abstract

**Background:**

Preterm infants are at greater risk for neurodevelopmental disabilities than full term infants. Interventions supporting parents to improve the quality of the infant’s environment should improve developmental outcomes for preterm infants. Many interventions that involve parents do not measure parental change, nor is it clear which intervention components are associated with improved parental outcomes. The aim of this review was to categorize the key components of early intervention programs and determine the direct effects of components on parents, as well as their preterm infants.

**Methods:**

MEDLINE, EMBASE, CINAHL, ERIC, and Cochrane Database of Systematic Reviews were searched between 1990 and December 2011. Eligible randomized controlled trials (RCTs) included an early intervention for preterm infants, involved parents, and had a community component. Of 2465 titles and abstracts identified, 254 full text articles were screened, and 18 met inclusion criteria. Eleven of these studies reported maternal outcomes of stress, anxiety, depressive symptoms, self-efficacy, and sensitivity/responsiveness in interactions with the infant. Meta-analyses using a random effects model were conducted with these 11 studies.

**Results:**

Interventions employed multiple components categorized as (a) psychosocial support, (b) parent education, and/or (c) therapeutic developmental interventions targeting the infant. All interventions used some form of parenting education. The reporting quality of most trials was adequate, and the risk of bias was low based on the Cochrane Collaboration tool. Meta-analyses demonstrated limited effects of interventions on maternal stress (*Z* = 0.40, *p* = 0.69) and sensitivity/responsiveness (*Z* = 1.84, *p* = 0.07). There were positive pooled effects of interventions on maternal anxiety (*Z* = 2.54, *p* = 0.01), depressive symptoms (*Z* = 4.04, *p* <.0001), and self-efficacy (*Z* = 2.05, *p* = 0.04).

**Conclusions:**

Positive and clinically meaningful effects of early interventions were seen in some psychosocial aspects of mothers of preterm infants. This review was limited by the heterogeneity of outcome measures and inadequate reporting of statistics.

**Implications of key findings:**

Interventions for preterm infants and their mothers should consider including psychosocial support for mothers. If the intervention involves mothers, outcomes for both mothers and preterm infants should be measured to better understand the mechanisms for change.

## Introduction

### Description of the condition

Preterm birth (before 37 complete weeks of gestation) is challenging for both the infant and the family. The long-term morbidity of these infants is a serious public health concern [1]. Organ systems are insufficiently developed to fully support extra-uterine life resulting in increased biological risk for complications of prematurity [2,3]. Compared to children born at term, preterm infants are at greater risk for neurodevelopmental disabilities including cerebral palsy [4], mental retardation [4,5], vision impairments [6,7], and hearing loss [4,8]. As preterm infants develop, there is an increased risk of cognitive and language delays [7,9,10], hyperkinetic disorders [4,11], behavioral and emotional problems [3,4], and learning disabilities [12-14]. These risks increase as gestational age at birth decreases [15-17]. The medical and educational services, and lost productivity associated with preterm infants cost more than $26.2 billion USD in 2005, or $51,600 per infant [18], and far exceed the costs for term infants [19,20]. Families caring for preterm infants experience increased stress [21-23], anxiety [21], and depression [21,24]. Together, these psychosocial factors influence maternal sensitivity and responsiveness in interactions with the infant [25], which ultimately affect child developmental outcomes [26].

Early interventions often target the child’s environment [27], which includes parents and assumes that a positive environment will subsequently improve child outcomes. The risk for developmental disorders varies by gestational age. Thus, the majority of interventions for preterm infants that involve parents are complex and include multiple components. While it is critical to understand how the intervention works [28], most evaluations do not explain how the key components exert their effect on parent or child outcomes. The **aim** of this review was to categorize the key components of early intervention programs for preterm infants and their parents, and determine their effects on parental stress, anxiety, depressive symptoms, self-efficacy, and sensitivity/responsiveness in interactions with the infant, and subsequently, on child development.

### How early interventions for preterm infants might work

For the purpose of this review, ‘early intervention’ refers to prevention-focused programs occurring soon after birth when the infant’s brain is plastic [29,30] and interventions are more likely to have maximal impact [30]. If efficacious early interventions are necessary to optimize outcomes for preterm infants, then clear evidence of the key components that contribute to these outcomes is required to refine complex interventions and target them to the preterm infants and families who will benefit most.

Theory guides the link between intervention components and outcomes. Psychosocial support of parents to decrease stress, anxiety, and depressive symptoms, and increase self-efficacy and maternal sensitivity and responsiveness in interactions with their infants should have a positive effect on the child’s environment [27], and subsequently improve the preterm child’s developmental outcomes. Educational intervention may increase parental knowledge and skill in caring for preterm infants and subsequently increase parenting self-efficacy and decrease stress. Education may include (a) information about infant growth and development, (b) demonstrations of infant behaviour with discussion, or (c) active involvement of the parent in interaction with the infant with feedback from a professional. Developmental intervention delivered by the parent may also have an effect on the child’s development. Thus, three main categories of intervention components may affect outcomes: psychosocial support for the parent, parenting education, and therapeutic developmental support for the child. What remains unclear in the literature is whether or not the intervention components have an effect on parents as the mechanism to improve outcomes for preterm infants. If so, it is unclear which components in complex interventions have the greatest effect on parents and development of preterm infants.

### Why is it important to do this review?

No other systematic review has examined the specific components of interventions with an effort to untangle the mechanisms underlying the effects on parent outcomes, and subsequently child outcomes. McCarton and colleagues [31] did a narrative review of a “representative sampling” (p. 331) of 19 programs dating back to 1971. They found only modest benefits for children with low birth weight (LBW). Infant-focused physiotherapy programs had little effect on motor or cognitive outcomes. Parent-focused programs improved the quality of parent-child interactions with inconsistent effects on cognitive development. Interventions with parent and child components had positive effects on parent-child interactions and child development. The authors concluded that interventions for preterm infants with parent support and education may provide a more optimal environment for child development. While McCarton and colleagues address parent outcomes, their review was not comprehensive, did not consider the quality of the studies, and requires updating.

The most recent systematic reviews and meta-analyses focused only on cognitive and motor outcomes of preterm infants [32-34]. In their Cochrane review of randomized controlled trials (RCTs) or quasi-RCTs of preventive early intervention programs for preterm infants up to 2009, Spittle et al. [35] and Orton et al. [32] found positive effects on preschool cognitive development that were not sustained at school age. There were no effects on motor development at any age. Vanderveen and colleagues [34] assessed RCTs or quasi-RCTs published up to 2008 of early interventions for preterm infants and parents. Intervention components were diverse and included education for parents, infant stimulation, home visitation, and developmental care in the neonatal intensive care unit (NICU). Meta-analyses of cognitive and motor development at 12 months showed significant improvements favouring the intervention groups. At 24 months, positive effects remained for cognitive scores only. By ages 3 and 5 years, no significant effects remained. A unique contribution of Vanderveen and colleagues’ review was the focus on parental involvement in care and recognition of diverse intervention components. While it is important to understand the effects on preterm infants, none of these reviews included outcomes for the parents who were targeted as the medium for delivery of the intervention.

Pridham and colleagues [36] conducted an integrative review of 22 nurse-led quantitative intervention studies designed to promote parent-child interactions and relationships. Preterm infants were not the specific focus, but some were included. These authors called for an increased emphasis on theoretical underpinnings of interventions with specific attention to factors that lead to child and parent outcomes. Ethnicity and parent gender (mothers and fathers) may influence interactions with young infants yet these variables are rarely studied. This knowledge gap was noted in a qualitative synthesis of parental perspectives on parent-child relationships by the same group [37] that included some studies of parents with preterm infants. Thus, important gaps in previous literature reviews exist, which need to be addressed.

The objectives of this systematic review of early interventions for preterm infants were to: (a) identify key parent outcomes, (b) determine the quality of evidence for the RCTs, (c) estimate the intervention effects on parent outcomes, (d) categorize the key intervention components associated with maternal outcomes and subsequently child outcomes, and (e) apply the results to clinical practice and future research in order to assist delivery of focused, cost-effective, preventive developmental interventions for preterm infants and their families.

## Methods

### Inclusion/exclusion criteria and definitions

Studies published between 1990 and 2011 were included if they met the following criteria: (a) published in English, (b) RCT, (c) children born preterm (gestational age less than 37 weeks), (d) primary RCT of a preventive intervention started before the child was age 3 years (corrected age); (e) intervention involved parents (mother and/or father), and (f) intervention included at least one session in the community (home or clinic). The critical importance of proximal environments for early child development led to our focus on interventions with at least partial home or community components. Multiple reports of the same sample were treated as a single study. Articles that reported a pilot RCT (i.e., Melnyk et al. [38]) and a full RCT (i.e., Melnyk et al. [39]) with different samples were treated as separate studies. Articles excluded from the review were (a) non-RCT designs, (b) review articles, (c) interventions occurred in hospital only, (d) focused on a subset of preterm infants such as those already diagnosed with a developmental problem such as a behavioural disorder, or (e) targeted nursing staff.

Drop outs were defined as non-completion of the intervention. Outcomes were defined as assessments prior to intervention and conducted again close to the end of the intervention. Follow-ups were assessments at any time point after the outcome assessment at completion of the intervention.

### Search strategy for identification of studies

#### Search strategy

A health sciences librarian (KAH) and investigators (KB and JME) developed the search strategy. Keywords and MeSH terms/subject headings were reviewed. Pretesting the search strategy and preliminary searches ensured that all appropriate keywords and subject headings were incorporated into the final search strategy. In order to focus on interventions including parents, MeSH/subject headings were used for the concept “parents” in every database searched. This strategy did result in one important study being missed initially [40], and was identified during hand searching of reference lists. The OVID MEDLINE final search strategy is reported in a supplementary Table.

Literature from 1990 to December 2011 was searched using the OVID interface for MEDLINE, EMBASE, and Cochrane Database of Systematic Reviews. CINAHL and ERIC were searched via the EbscoHost interface. The search strategy was saved for each database so it could be re-run to update the search. The year 1990 was selected to align with the implementation of developmental care [41] and increased parental involvement in the care of preterm infants. The reference lists of each included article were hand searched. Most of the references were additional reports of the RCTs that had already been identified or additional follow-up studies. Some references pre-dated 1990. Tables of Contents were not searched because of the magnitude of a search of multiple journals from diverse disciplines over the established timeline. The Web of Science was used to determine ‘cited bys’. No additional articles were identified.

### Data collection and analysis

#### Data extraction and management

Searches were exported to separate folders in the bibliographic management program RefWorks [42] and later merged into a single folder. Duplicates were deleted. Data were entered into RevMan 5.1 [43] for analyses.

#### Assessment of bias in included studies

Methodological quality was assessed by MB and KB using the Cochrane Collaboration’s Tool for assessing risk of bias [44]. After discussion, there was complete agreement between MB and KB on the risk of bias scores. See supplementary table, additional file 1 . Publication bias was assessed using funnel plots with standard error as the vertical axis and standard mean difference for intervention effects on the horizontal axis [45].

#### Measurement of treatment effect on parental stress, anxiety, depressive symptoms, self-efficacy, and sensitivity/responsiveness

In studies that included parental outcome scores, standardized scales were used. The principal summary measures were means and standard deviations. Standardized mean differences were calculated because there were multiple outcome measures with different standard deviations. Pooled effects for treatment were calculated using a random effects model because of large heterogeneity (i.e., *I*^2^) across studies overall.

#### Unit of analysis issues

Although the term parent was used in the majority of studies, the unit of analysis was the mother. Therefore, only information related to mothers is reported.

#### Effect size analyses

Effect sizes were included for continuous level outcomes from the measures of stress, anxiety, depressive symptoms, self-efficacy, and sensitivity/responsiveness.

No corrections were made to account for studies with small samples. The value for the test of significance for overall effect was set at *p* < .05.

#### Assessment of heterogeneity of the studies

Heterogeneity was assessed using *I*^2^ statistic. Studies in the review varied in terms of sample size, number and type of intervention components, delivery format, and outcome measures. A meta-analysis was conducted when an outcome was reported in two or more studies. No subgroup analysis was planned or conducted.

### Results of the search

**Figure 1:**
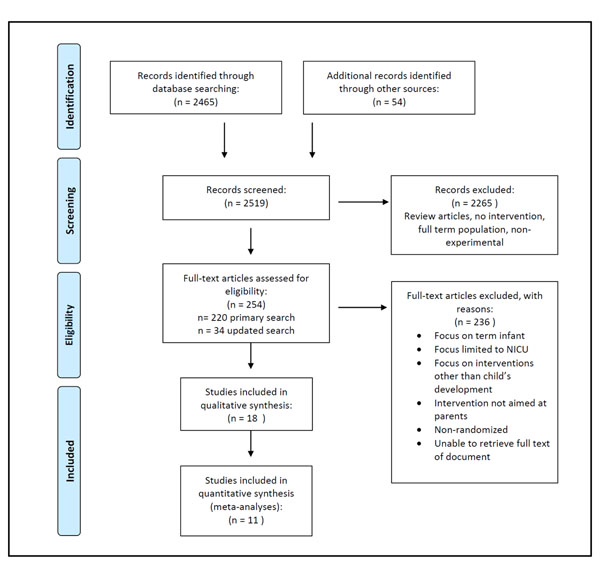
Study selection flow diagram

The primary literature search yielded 2465 titles and abstracts, of which 220 were identified by KB and KAH as potentially relevant for full text review. Inter-rater agreement was 99% between KB and KAH on the first 100 titles and abstracts from Medline extracted to August 10, 2010. Disagreements were resolved by consensus. Upon critical review of the full text, only 19 articles met the inclusion criteria. Upon detailed review, the study by Sajaniemi et al. [46] was excluded because it was a case control study. Bagner et al. [47] was excluded because the intervention targeted older (range 18 to 60 months) preterm children with a diagnosed behavioural disorder. The search was updated on December 31, 2011 and yielded 34 additional titles and abstracts. From these, one additional RCT [48] was added. Thus, 18 studies were included in this review. See Figure 1 for the study selection flow diagram.

### Description of studies

#### Study characteristics

The included studies were conducted over the last 21 years; 45% of studies were published in the last 5 years. Studies were from Australia [49,50]; England [51,52]; Germany [53]; Italy [54]; Japan [55]; Netherlands [56]; Norway [23]; and the United States [38,40,57-61]. See Table 1 for characteristics and methods of studies included in the review. One study reported a cluster RCT [52]. All studies included preterm infants in an intervention group (e.g., kangaroo care [58]) that was compared to a control (e.g., standard care) or comparison group (e.g., traditional holding [58]). A few studies included a term reference group. Preterm infants were matched on demographic variables to a term reference population born near the same time [51], while Kaaresen et al. [23] included a non-matched term reference group. Follow- up studies were published for six RCTs [23,40,50-52,62], and follow-up ranged from 6 months [56] to 18 years [63] after birth.

**Table 1:** Characteristics and methods of studies included in review (by date).

Study	Study quality*	Infants enrolled	Drop outs	No. of sites	Mean GA (weeks)		Mean birth weight (grams)		Group allocation		Follow-up
		*n*	*n* (%)		I	C	I	C	I (*n*)	C (*n*)	

IHDP 1990	4	985	72(7.3)	8	33.0	33.0	1819	1781	377	608	18 yrs
Kang 1995	2	327	82(25.1)	3	33.1^g^	33.1^g^	1938^g^	1938^g^	197	130	5 m
Saylor 1996	3	68	3(4.4)	1	28.9	30.3	1107	1231	34^f^	31^f*^	7 yrs
APIP 1998	4	328	42(12.8)	2	31.0 ^d^ P 30.0^d^ PA	31.0^d^	1560 P 1331 PA	1420	116 P 106 PA	106	5 yrs
Melynk 2001	2	55	13(23.6)	1	31.4	31.6	1483	1731	26	29	6 m
Brisch 2003	2	87	11(12.6)	1	27.0	27.0	919	987	43	44	24 m
Ohgi 2004	3	23	1(4.2)	1	30.3	30.3	1273	1360	12	11	6 m
Gianni 2006	2	36	0(0)	1	28.3	27.5	892	836	18	18	36 m
Kaaresen 2006	4	146 75^a^	7(3.2)	1	30.2	29.9	1402	1393	72	74 75^b^	5 yrs
Melynk 2006	3	260	13(5.8)	2	31.3	31.4	1683	1627	147	113	2 m
Glazebrook 2007^b^	3	233	11(4.7)	6	28.5^d^	29.0^d^	1121	1220	112	121	24 m
Koldewijn 2009	3	176	1(0.6)	7	29.6	30.0	1242	1306	86	90	7 yrs
Newnham 2009	3	68	5(7.4)	1	31.3	33.7	1590	1619	35	33	24 m
Teti 2009	3	194	21(10.8)	4	30.6	30.0	1455	1397	99	95	4 m
Milgrom 2010	4	45	0(0)	1	27.5	27.7	981	999	22	23	0
Neu 2010	2	87	8(9.2)	5	33.1	33.4^e^ 33.4^e^	1990	1880 1980	31	29^e^ 27^e*^	4 m
Spittle 2010	4	120	3(2.5)	2	27.3	27.4	1029	991	61	59	24 m
Ravn 2011	4	118	0(0)	1	33.3	33.2	1935	1919	61	57	12 m

*Note:* *Study methodological quality was assessed by Cochrane Collaboration’s Tool [44].

GA = gestational age; I = Intervention group; C = Control or comparison group; m = months; yrs = years

Programs: APIP = Avon Premature Infant Project; IHDP = Infant Health Development Program; P = Portage intervention; PA, = Parent Advisor intervention

^a^ Matched term controls

^b^ Cluster RCT with washout and crossover

^c^ Case matched by developmental risk score, then randomly allocated to groups

^d^ Median

^e^ Holding comparison group; ^e*^ Control group

^f^ Early intervention group; ^f*^ Late intervention group

^g^ Overall mean for cohort

#### Intervention participants

All studies involved interventions that included mothers, yet seven of the 18 studies measured only child outcomes [40,51,53,54,58,60,64]. While these studies make important contributions to understanding the effects of interventions on preterm infants, they do not contribute directly to an understanding of the differential effects on parents (i.e., mothers and fathers). Five studies included fathers in their interventions [23,39,50,53,62], but only Kaaresen et al. [23] and Melnyk et al. [39] reported outcomes for them. In one study, only one father participated [50], and in another [53], data for fathers were not collected. Koldewijn et al. [62] described the proportion of fathers who participated in the intervention. Outcomes for mothers were reported only in their follow up studies [56,65,66].

The infants were described as preterm (< 37 weeks gestation) and/or low birth weight (< 2500 g). Saylor and colleagues [60] targeted preterm infants with an intra-ventricular hemorrhage and also randomized preterm infants to early versus late start of the intervention. Inclusion of singleton versus multiple births varied across studies. Four studies [38,39,54,55] included only singleton births. Five studies [40,57,58,60,61] included mother-infant pairs or dyads without mention of multiple gestations. In studies that reported allocation of multiple gestations [23,53], infants from one family were assigned to the same group. Three studies that included multiple gestations [23,48,51] did not report how they managed twins and triplets in their analyses. Koldewijn et al. [62] reported that one child per family was selected for analyses; Brisch et al. [53] reported data from the first born twin; and Newnham et al. [49] and Milgrom [64] calculated sibling averages. Only Glazebrook et al. [52] and Spittle et al. [50] adequately controlled for multiple gestations by clustering.

#### Sample sizes

The sample sizes varied from 23 [55] to 985 [40]. Recruitment was problematic for three studies [50,55,58] with two studies [50,55] terminated without achieving the planned sample size. One program targeted an ethnically homogeneous population (i.e., African American; Teti, [61]); however, the majority of studies did not report ethnicity.

#### Quality of the evidence

The methods to generate the randomization sequence and prevent subversion were adequately described and there was a low risk of bias related to random sequence generation in 70% of the trials. Only four studies [23,51,64,67] adequately described the concealment of treatment allocation. By their nature, most were single blind studies with outcome assessors only blind to study group. Only Melnyk et al. [38,39] concealed group allocation by distributing information in identical envelopes. Several studies had high refusal rates that ranged from 45.2% [39] to approximately 60% [53,58]. Attrition was high in two studies, with losses of 23.6% [38] and 24% [57]. Overall, the reporting quality of the studies was adequate. The strongest studies according to the quality rating scale (Cochrane Collaboration’s Tool [44]) are indicated in Table 1[23,48,50,51,61,67].

#### Publication bias

Visual inspection of funnel plots suggested effect sizes for the studies reporting outcomes for stress, anxiety, depressive symptoms, self-efficacy, and sensitivity/responsiveness were scattered symmetrically around a central effect. These analyses were limited by the small number of heterogeneous studies with diverse outcome measures. Thus, the results of the assessment of publication bias should be treated with caution.

#### Intervention components

While most studies provided some description of the intervention components, few reported sufficient detail to enable replication. Except for Gianni et al. [54], most delivered an intervention with a curriculum or activities defined in a user’s manual. Only studies by Melnyk et al. [38,39] included details about timing and number of sessions, and program staff education and training.

The interventions reviewed had multiple and diverse components, and a coding system was needed to group interventions. Egeland et al. [68] coded four types of intervention approaches that promote positive change in interactions. These approaches (a) promoted parental awareness of the child, interpretation of child behaviours, and responsiveness to the child, (b) provided support to the parent including advice, anticipatory guidance, and/or emotional support, (c) increased parental awareness of their influence on their child, or (d) promoted parental well-being through decreased stress. Bakersmans-Kranenburg et al. [69], in their meta-analysis, integrated parental well-being into the first three of Egeland and colleagues’ [68] categories. Based on a bio-ecological framework, we coded intervention components into three broad categories: (a) parent support (i.e., psychological counseling and social support), (b) parent education (i.e., information, demonstration and discussion, and active engagement with feedback from a professional), and (c) therapeutic child development support. KB and MB assessed the components reported in each study. After discussion, there was complete agreement about codes for the intervention components. See Table 2 for the intervention components and coded categories for each study.

Some aspect of parenting education was integral to all interventions. One study provided all three intervention components [67]. Eight studies provided parent support and parenting education [23,48-51,53,54,64], while two interventions combined parenting education with therapeutic child development support [60,62]. The remaining seven interventions [38,39,52,55,57,58,61] provided parenting education only.

**Table 2:** Intervention components, coded categories, and maternal and child development outcomes

Study	Name of program	Intervention components					Maternal outcomes		Child developmental outcomes	
		Parent support	Parent education categories			Child support	Short-term < 1 yr	Long-term > 1 yr	Short-term < 1 yr	Long-term > 1 yr
			1	2	3					

Higher quality studies ≥ 3*										

IHDP 1990	IHDP	+	+	-	-	+	n.a.	-	**-**	**+**
Saylor 1996	CAMS	-	+	+	+	+	-	-	**-**	-
Avon 1998	APIP	+	+	-	-	-	n.a.	n.a.	n.a.	-
Ohgi 2004	NBAS based	-	+	+	-	-	+	n.a.	-	n.a.
Kaaresen 2006	MITP Modified	+	-	+	+	-	+	+	+	+
Melynk 2006	COPE	-	+	-	-	-	+	n.a.	n.a.	n.a.
Glazebrook 2007	PBIP	-	+	+	+	-	-	n.a.	-	-
Koldewijn 2009	IBAIP	-	+	-	+	+	n.a.	n.a.	+	+
Newnham 2009	Modified MITP	+	+	+	+	-	+	+	n.a.	+
Teti 2009	NBAS based	-	+	+	+	-	+	n.a.	+ ELBW only	n.a.
Milgrom 2010	Modified MITP	+	+	+	+	-	n.a.	n.a.	+	n.a.
Spittle 2010	VIBeS Plus	+	+	+	-	-	n.a.	+	+	+
Ravn 2011	MITP	+	+	-	+	-	+	n.a.	n.a.	n.a.

Lower quality studies (< 3)*										

Kang 1995	NSTEP-P	-	+	+	-	-	+	n.a.	+	n.a.
Melynk (pilot) 2001	COPE	-	+	-	-	-	-	n.a.	+	n.a.
Brisch 2003	n.a	+	-	-	+	-	n.a.	n.a.	n.a.	-
Gianni 2006	n.a	+	-	+	-	-	-	n.a.	-	-
Neu 2010	Kangaroo Care	-	+	-	+	-	+	n.a.	n.a.	n.a.

*Note*: * Quality determined by Cochrane Collaboration’s Tool ; n.a. = not assessed or not reported; ELBW = extremely low birth weight infant

**Coding categories for parent education components:** 1 = **Information given**: Included information that is generic (e.g., written, audiotape or videotape format), individualized to the family (e.g., written or verbal), and/or included discussion with the parent; 2 = **Guided observation**: included parent observation or demonstration of an activity with the infant; 3 = **Active involvement**: included parent involvement in active practical experiences, modeling and guided self-evaluation or self-reflection learning (e.g., video feedback).

**Names of Intervention Programs:** APIP = Avon Premature Infant Project; CAMS = Curriculum and Monitoring System; COPE = Creating Opportunities for Parent Empowerment NICU Program; IBAIP = Infant Behavioral Assessment and Intervention Program; IHDP = Infant Health and Development Program; NBAS = Neonatal Behavioral Assessment Scale, Brazelton; NSTEP-P = Nursing Systems for Effective Parenting-Preterm; MITP = Maternal Infant Transaction Program; PBIP = Parent Baby Interaction Program; PCIT = Parent Child Interaction Therapy; Premie Start = Modification of MITP; SI- NDT = Sensory Integration and Neurodevelopmental Therapy; SM=State Modulation; VIBeS Plus = Victoria Infant Brain Studies.

The parenting education component was further divided into (a) information only (generic or individualized to the family, and may include discussion of information); (b) guided observation of the infant, or (c) active involvement of parent in learning about their infant and guided reflection or self-evaluation. Four studies used information only [38-40,51]. One study used only guided observation of the infant [54], and one used active involvement with the infant [53]. Five studies used all three types of parenting education [49,52,60,61,64]. Three interventions combined information with guided observation [50,55,57], and three combined information and active involvement [48,58,62]. Only Kaaresen et al. [23] combined guided observation with active involvement.

### Synthesis of results

Eleven of the 18 included studies reported maternal outcomes of stress, anxiety, depressive symptoms, self-efficacy, and sensitivity/responsiveness in interactions with the infant. These 11 studies [23,38,39,48-50,52,55,57,61,62] were used in the synthesis of parent outcomes. Other parent outcomes were measured, but only one study addressed each additional construct so effect sizes were not calculated for those constructs (e.g., [58]). Data were not provided to calculate the mean and standard deviation in two studies [58,60]. Seven studies did not measure parent outcomes [40,51,54,58,60,64]. Two studies were missing data required to calculate effect sizes. Melnyk et al. [39] reported means, confidence intervals, and sample sizes (N). Standard deviations (SD) were computed using the following formula assuming standard error (SE) for 95% confidence intervals = 1.96:

Means and standard deviations were calculated from frequencies and percentages for scores reported by Ravn et al. [48]. Saylor et al. [60] reported correlations only and means/standard deviations could not be calculated; therefore, it was not included in the meta-analysis.

#### Stress

**Figure 2:**
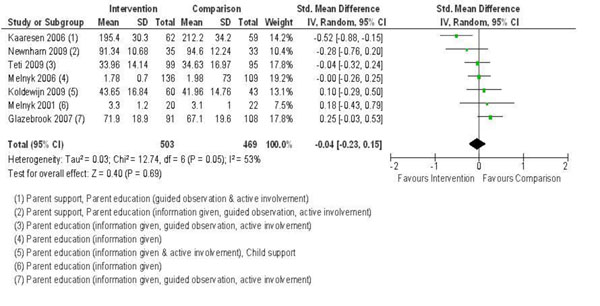
Forest plot of comparison: 1 Group, outcome: 1.1 Stress.

Stress was measured in seven studies. Five studies [23,49,52,61] reported various subscales and total scores of the Parenting Stress Index [70] long or short form. Koldewijn et al. [62] did not report parenting stress in their initial report of the RCT, but did in the 12 and 24 month follow-up study [65]. Melnyk [38,39] measured stress using the Parental Stressor Scale: NICU [71]. Pooled effects are reported in Figure 2. The test for overall effect of the interventions on stress was not significant. Kaaresen et al. [23] and Newnham et al. [49] demonstrated the greatest decrease in stress with an intervention that included parent support and education approaches (including guided observation and active involvement). There were positive child cognitive outcomes in Kaaresen et al.’s follow-up study of 5-year-olds [72], and positive effects on temperament for Newnham et al. [49] at 3 and 6 months. Focusing only on parent education [38,39,52,61], or parent education and child developmental support [62] had no effects on parental stress.

#### Anxiety

**Figure 3:**
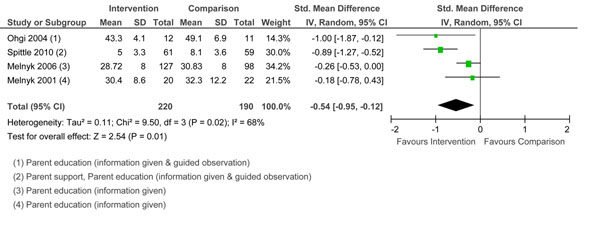
Forest plot of comparison: 1 Group, outcome: 1.3 Anxiety.

Anxiety was measured in four studies. Three studies used the State-Trait Anxiety Inventory– State score [73]. Spittle et al. [50] used the Hospital Anxiety and Depression scale [74]. Pooled effects are reported in Figure 3. There was a significant reduction in anxiety across studies. Interventions with the greatest reduction in anxiety included two types of parenting education (information and guided observation; [50,55]) with Spittle et al. [50] including parent support. Ohgi et al. [55] included only 24 mothers with large variability in anxiety scores. Studies with positive effects on anxiety showed positive effects on child development over the short term [38,55] and up to 24 months [50].

#### Depressive symptoms

**Figure 4:**
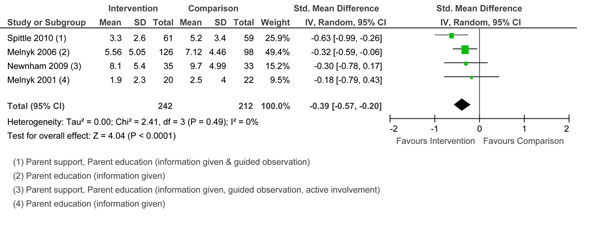
Forest plot of comparison: 1 Group, outcome: 1.2 Depressive symptoms.

Four studies measured depressive symptoms, each using a different measure. Pooled effects are reported in Figure 4. The test for overall effect was significant. It is unclear which intervention components contributed to the decrease in depressive symptoms. Melnyk et al. in their studies [38,39] provided parent education (information only), and demonstrated similar positive effects to studies by Spittle et al. [50] and Newnham et al. [49] that provided more complex parent education as well as parent support. Studies with positive effects on depressive symptoms showed positive effects on child outcomes over the short term [38,39] and up to 24 months [49,50].

#### Self-efficacy

**Figure 5:**
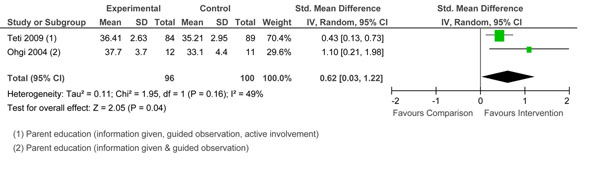
Forest plot of comparison: 1 Group, outcome: 1.4 Self-Efficacy.

Two studies [55,61] used different measures of self-efficacy. Pooled effects are reported in Figure 5. The overall effect was significant. The interventions used two or three types of parenting education. Both studies resulted in improved child outcomes over the short term only. Only Teti et al.[61] demonstrated positive child cognitive outcomes at 4 months.

#### Sensitivity/responsiveness

**Figure 6:**
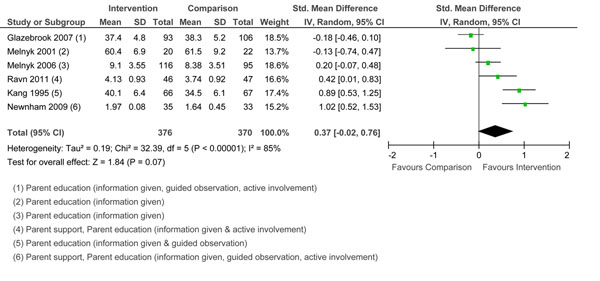
Forest plot of comparison: 1 Group, outcome: 1.5 Sensitivity/Responsiveness.

The meta-analysis included six studies that measured sensitivity/responsiveness. Three studies used various subscales and total scores of the Nursing Child Assessment Satellite Training teaching and/or feeding scales [75]. Others [67] used qualitative ratings from a sensitivity/responsiveness subscale [76]. Pooled effects are reported in Figure 6. The test for overall effect was not significant. Two of the studies [48,49] that showed a positive effect on maternal sensitivity/responsiveness included parenting education with active involvement and parent support. Interventions that provided information only [38,39] were less effective. The Glazebrook et al. program [52] was not effective although they used all three types of parenting education in their intervention. Studies that did not show an effect of the intervention on maternal sensitivity/responsiveness showed mixed effects on child outcomes [38,52]. Studies that showed a positive effect on maternal sensitivity/responsiveness were associated with positive infant mood at 12 months [48] and positive temperament [49]. In assessments of sensitivity/responsiveness, it is difficult to untangle the effects of the parent and child contributions to interactions.

## Discussion

As a result of early interventions for mothers of preterm infants, positive and clinically meaningful effects were seen for anxiety, depressive symptoms, and self-efficacy. To our knowledge, this review was the first to categorize components of early interventions and link them to maternal outcomes. These components were parent psychosocial support, parenting education, and therapeutic developmental support for the infant. Some form of parenting education was used in all interventions. Interventions that included psychosocial support resulted in better outcomes for mothers of preterm infants.

There were overall positive effects of four interventions on maternal anxiety with improved child outcomes. Anxiety is an important construct to measure in mothers of preterm infants [21]. The results suggest that it may be more consistently linked to child outcomes than is stress. Parenting education may be a key intervention component associated with decreasing anxiety although all 18 interventions provided some form of education with varying effects on other parent constructs. Only one of the studies that had an effect on anxiety included a parent support component so this component may be a less important aspect of intervention when addressing anxiety.

Three interventions, two with a parent support component, had positive effects on maternal depressive symptoms. The Victoria Infant Brain Studies [50] and Creating Opportunities for Parent Empowerment [38,39] were effective for depressive symptoms as well as anxiety. The Mother Infant Transaction Program [23,48,49,64] also had an impact on stress. Given that depression is a common and costly problem for mothers of preterm infants [21,24], it should be measured consistently as an outcome measure of the effectiveness of interventions. Depressive symptoms for mothers of preterm infants stem from multiple factors (e.g., high perceived stress and low social support) [77] and therefore multiple intervention approaches that reduce stress and increase social support are required to reduce the development of depressive symptoms.

Only two studies measured self-efficacy and both found positive effects. One study [55] had a very small sample, and the other [61] included only African American mothers, limiting the quality and generalizability of the evidence. The positive effects of the interventions on self-efficacy were associated with improved infant outcomes only over the short-term. Intuitively this makes sense because the two interventions used information combined with observation of the infant or active involvement with the infant. Information may be relevant at the time it is delivered, but infants change qualitatively over time and additional information sessions may be important to achieve sustained effects on self-efficacy. Alternatively, interventions for self-efficacy may have limited effects on long term outcomes for preterm infants.

The second most commonly measured construct for parent outcomes was sensitivity/responsiveness and the overall effect was not significant. This construct is difficult to capture as it depends on the child’s skills as well as the parents. Intervention can target only the parent side of interactions. For the three studies that found an increase in sensitivity/responsiveness, two of the interventions included parent support as well as parenting education. All three of the studies that showed little or no change had no parent support but several types of parenting education. Despite Pridham and colleagues’ [36] integrative review of nursing interventions that encouraged parenting education and discussion of child behaviour, it would appear that more than just education is needed. In addition, Newnham et al. [49] suggested that aspects of parent-child interactions with preterm infants may be more challenging at different ages, thus time at outcome measurement may be critical in measuring sensitivity/responsiveness. Location for interventions (NICU and home) may influence outcomes that target parent-child interaction resulting in inconsistent outcomes [56].

Stress was the most commonly measured construct of all maternal psychosocial outcomes. This is not a surprise given that the literature has reported increased stress for families caring for preterm infants [21-23]. However, the meta-analysis suggests that the interventions reviewed have little effect on stress overall. It was not clear why there were limited effects on stress overall when the seven studies were considered together. The two interventions with an effect on stress [23,49] used a parent support component combined with an active involvement type of education and also found positive child outcomes. Parenting education alone did not reduce stress. The five studies with little or no effect did not have a parent support component, which may be important for addressing stress. The Parenting Stress Index [70] was often used allowing comparison across studies but it may be too generic a measure of stress for mothers of preterm infants. Various subscale and total scores of the short and long forms of the measure were used making it difficult to untangle which aspects of parenting stress are of concern for mothers of preterm infants. Alternatively, it may be difficult for interventions to reduce stress by the time of outcome measurements. Kaaresen et al. [23] measured stress three months after the completion of the intervention and reported reduced stress levels and improved long-term child outcomes. In most studies, stress was measured at completion of the intervention [38,39,49,61] or within 6 weeks of the intervention [52]. Studies that used the Parental Stressor Scale- NICU also failed to show positive effects on stress at NICU discharge, a point in time when parent stress levels are reportedly very high [21,78]. The time point at which the stress outcome was assessed may influence the apparent effectiveness of interventions. Alternatively, interventions may have different effects on parental stress depending on severity of infant illness. Timing of measurement and subgroup analyses should be considered in future research.

It is clear that no one intervention component is consistently associated with improved parent outcomes. The interventions provided were complex with multiple and varying components. Indeed, parent education was the only component used in all of the interventions and the delivery, type and dose of parenting education varied. Of the interventions that provided direct support to parents, only half measured outcomes for parents. It is encouraging that the studies that provided support found positive effects on parent outcomes that were often associated with improved child outcomes. Parent support may be an important component of interventions for preterm infants.

This review is limited by several factors, such as inclusion of studies published in English only. Inclusion of studies published in other languages may alter the results of meta-analyses. Lack of socio-demographically homogeneous samples (except Teti et al. [61]) may have influenced the ability to demonstrate effects of the interventions on mothers and preterm infants. One study [50] had challenges recruiting sufficient sample and was underpowered. Positive effects may not have been identified. The measures used in the studies included in the meta-analyses varied and the results may reflect differences in the construct being measured. It was also not possible to consider the effects of dose, location of the intervention, or study quality when conducting the meta-analyses. Finally, the I^2^ statistic suggested significant heterogeneity among the studies in the synthesis. Even using a random effects model, heterogeneity is a limitation in this review.

A problem with many studies in this review was that they reported “caregiver” (e.g., Meijssen et al, [56]) or “parent” and included data for mothers, only. Other studies (e.g., Spittle [50]) reported 98% female caregivers without indicating the relationship of the remaining 2% of caregivers to the child. If those caregivers were fathers, then they should be analyzed separately or excluded from analyses because mother-infant and father-infant interactions are different [79]. Given the small number of fathers who participated in interventions, it is unlikely that this would have had a substantial effect on the overall results. A further limitation of this review is that the key components of the interventions can only be interpreted based upon what the authors of the trials have published. It is possible that our interpretations of the interventions may not reflect what has occurred due to lack of detail regarding intervention content in the reviewed studies.

Our most important recommendation for future research is to measure the effects of intervention components addressing parents to determine whether parent outcomes change, thus altering the child’s proximal environment. One third of the studies in this review did not directly and systematically measure parent outcomes. Consistent measures of parent outcomes would enhance the ability to build the knowledge base. Another suggestion relates to fathers. Spittle et al. [33] and Orton et al. [32] noted the lack of evidence-based approaches to evaluate the effectiveness of interventions for fathers. There is a critical need to conduct more rigorous intervention research in the area of interventions for fathers generally, and fathers of preterm infants specifically. Other directions for future research include the content of parenting education. Given the state of science in early brain development, promoting the development of executive functioning, a developmental problem for many preterm infants [80], is important. In addition, the most efficient and effective manner of delivering parenting education is still unclear. Five studies [49,52,60,61,64] used all three types of parenting education with varying effects on parent outcomes. Others [38,39,51] used relatively minimal forms of parenting education (information only) and found effects on parent outcomes. Unmeasured co-morbidities that would affect outcomes for preterm infants should also be captured. For example, Olafsen et al. [81] address regulatory competence which represents early aspects of communication and joint attention. Thus, regulatory competence may be important to measure as a useful precursor to language development. Finally, the use of other statistical techniques that can model moderators and mediators of the intervention effects on outcomes (e.g., structural equation modeling) may be useful.

## Conclusions

Results from this review provide the foundation for developing and testing interventions for parents of preterm infants. Thoughtful development of large, multi-site RCTs that could test multiple components of interventions including components that show promise in improving parental and child outcomes may be useful. This review is an effort to better understand the mechanisms underlying interventions for preterm infants that involve parents. Future research needs to focus on better understanding of components of intervention and their effects on child outcomes to ensure that resources can be targeted to achieve the greatest return on investment. Direct parent support is one component to consider in future research.

## Abbreviation

NICU: neonatal intensive care unit.

## Competing interests

The authors declare that they have no competing interests.

## Authors’ contributions

KB, JM, and KAH were responsible for the study conception and design. KAH designed the database search strategies. KB and KAH reviewed articles for relevance and performed critical appraisal. MB critically appraised relevant articles. KB wrote the first draft of the manuscript. KB, JM, KAH, and MB made critical revisions to the manuscript to improve intellectual content. KB and JM obtained the funding. All authors read and approved the final version.

## Supplementary Material

Additional File Supplementary Table - Final search strategy Click here for file

## Declarations

This article has been published as part of *BMC Pregnancy and Childbirth* Volume 13 Supplement 1, 2013: Preterm Birth: Interdisciplinary Research from the Preterm Birth and Healthy Outcomes Team (PreHOT). The full contents of the supplement are available online at http://www.biomedcentral.com/bmcpregnancychildbirth/supplements/13/S1

All of the publication fees will be funded by the Preterm Birth and Healthy Outcomes Team Interdisciplinary Team Grant (#200700595) from Alberta Innovates - Health Solutions, formerly the Alberta Heritage Foundation for Medical Research.

## References

[B1] Raju TNK Higgins RD Stark AR Leveno KJ Optimizing care and outcome for late-preterm (near-term) infants: A summary of the workshop sponsored by the National Institute of Child Health and Human Development Pediatrics 2006 118 3 1207 10.1542/peds.2006-0018 16951017

[B2] Anderson P Doyle L Neurobehavioral outcomes of school-age children born extremely low birth weight or very preterm in the 1990s The Journal of the American Medical Association 2003 289 24 3264 3272 10.1001/jama.289.24.3264 12824207

[B3] Bhutta AT Cleves MA Casey PH Cradock MM Anand KJ Cognitive and behavioral outcomes of school-aged children who were born preterm: A meta-analysis Journal of the American Medical Association 2002 288 6 728 737 10.1001/jama.288.6.728 12169077

[B4] Moster D Terje R Markestad T Long-term medical and social consequences of preterm birth N Engl J Med 2008 359 3 262 273 10.1056/NEJMoa0706475 18635431

[B5] Petrini J Dias T McCormick M Massolo M Green N Escobar GJ Increased risk of adverse neurological development for late preterm infants J Pediatr 2009 154 169 176 10.1016/j.jpeds.2008.08.020 19081113

[B6] Baron IS Erickson Kb Ahronovich MDK Coulehan K Baker R Litman FR Visuospatial and verbal fluency relative deficits in ‘complicated’ late-preterm preschool children Early Hum Dev 2009 85 12 751 754 10.1016/j.earlhumdev.2009.10.002 19879072

[B7] Pietz J Peter J Graf R Rauterberg-Ruland I Rupp A Sontheimer D Linderkamp O Physical growth and neurodevelopmental outcome of nonhandicapped low-risk children born preterm Early Hum Dev 2004 79 2 131 143 10.1016/j.earlhumdev.2004.05.001 15324993

[B8] Marret S Ancel P Marpeau L Marchand L Pierrat V Larroque B Foix-L'Helias L Thiriez G Fresson J Alberge C Neonatal and 5-year outcomes after birth at 30-34 weeks of gestation Obstet Gynecol 2007 110 1 72 80 10.1097/01.AOG.0000267498.95402.bd 17601899

[B9] Magill-Evans J Harrison MJ Parent-child interactions, parenting stress, and developmental outcomes at 4 years Child Health Care 2001 30 2 135 150 10.1207/S15326888CHC3002_4

[B10] Jennische M Sedin G Spontaneous speech at 6 1/2 years of age in children who required neonatal intensive care in 1986-1989 Acta Paediatr 2001 90 1 22 33 10.1080/080352501750064833 11227328

[B11] Linnet KM Wisborg K Agerbo E Secher NJ Thomsen PH Henriksen TB Gestational age, birth weight, and the risk of hyperkinetic disorder Arch Dis Child 2006 91 655 660 10.1136/adc.2005.088872 16754656 PMC2083047

[B12] Huddy CLJ Johnson A Hope PL Educational and behavioural problems in babies of 32–35 weeks gestation Arch Dis Child Fetal Neonatal Ed 2001 85 1 F23 F28 10.1136/fn.85.1.F23 11420317 PMC1721280

[B13] Kirkegaard I Obel C Hedegaard M Henriksen TB Gestational age and birth weight in relation to school performance of 10-year-old children: A follow-up study of children born after 32 completed weeks Pediatrics 2006 118 4 1600 1606 10.1542/peds.2005-2700 17015552

[B14] Chyi L Lee H Hintz S Gould JB Sutcliffe T School outcomes of late preterm infants: Special needs and challenges for infants born at 32 to 36 weeks gestation J Pediatr 2008 153 1 25 31 10.1016/j.jpeds.2008.01.027 18571530

[B15] McCormick MC Brooks-Gunn J Workman-Daniels K Turner J Peckham GJ The health and developmental status of very low-birth-weight children at school age Journal of the American Medical Association 1992 267 16 2204 2208 10.1001/jama.1992.03480160062035 1556798

[B16] Tomashek KM Shapiro-Mendoza C Davidoff MJ Petrini J Differences in mortality between late-preterm and term singleton infants in the United States, 1995-2002 J Pediatr 2007 151 450 456 10.1016/j.jpeds.2007.05.002 17961684

[B17] Sun Y Hsu P Vestergaard M Christensen J Li J Olsen J Gestational age, birth weight, and risk for injuries in childhood Epidemiology 2010 21 5 650 657 10.1097/EDE.0b013e3181e94253 20585254

[B18] Beck S Wojdyla D Say L Betran AP Merialdi M Requejo JH Rubens C Menon R Van Look PFA The worldwide incidence of preterm birth: A systematic review of maternal mortality and morbidity Bull World Health Organ 2010 88 1 31 38 10.2471/BLT.08.062554 20428351 PMC2802437

[B19] Clements KM Barfield WD Ayadi MF Wilber N Preterm birth-associated cost of early intervention services: An analysis by gestational age Pediatrics 2007 119 4 e866 e874 10.1542/peds.2006-1729 17339387

[B20] Wang ML Dorer DJ Fleming MP Catlin EA Clinical outcomes of near-term infants Pediatrics 2004 114 2 372 376 10.1542/peds.114.2.372 15286219

[B21] Singer LT Salvator A Guo S Collin M Lilien L Baley J Maternal psychological distress and parenting stress after the birth of a very low-birth-weight infant Journal of the American Medical Association 1999 281 9 799 805 10.1001/jama.281.9.799 10071000 PMC10189739

[B22] Singer LT Davillier M Bruening P Hawkins S Yamashita TS Social support, psychological distress, and parenting strains in mothers of very low birthweight infants Family Relations 1996 45 3 343 350 10.2307/585507 PMC4244282 25431508

[B23] Kaaresen PI Ronning JA Ulvund SE Dahl LB A randomized, controlled trial of the effectiveness of an early-intervention program in reducing parenting stress after preterm birth Pediatrics 2006 118 1 e9 10.1542/peds.2005-1491 16818541

[B24] Miles MS Holditch-Davis D Schwartz TA Scher M Depressive symptoms in mothers of prematurely born infants J Dev Behav Pediatr 2007 28 1 36 44 10.1097/01.DBP.0000257517.52459.7a 17353730

[B25] Field T Postpartum depression effects on early interactions, parenting, and safety practices: A review Infant Behavior and Development 2010 33 1 6 10.1016/j.infbeh.2009.10.005 19962196 PMC2819576

[B26] Forcada-Guex M Pierrehumbert B Borghini A Moessinger A Muller-Nix C Early dyadic patterns of mother-infant interactions and outcomes of prematurity at 18 months Pediatrics 2006 118 1 e107 e114 10.1542/peds.2005-1145 16818525

[B27] Bronfenbrenner U Bronfenbrenner U The biological theory of human development Making human beings human: Biological perspectives on human development 2005 Thousand Oaks, CA: Sage Publications 3 15

[B28] Craig P Dieppe P Macintyre S Mitchie S Nazareth I Petticrew M Developing and evaluating complex interventions: the new Medical Research Council guidance BMJ 2008 337 a1655 10.1136/bmj.a1655 PMC2769032 18824488

[B29] Katusic A Early brain injury and plasticity: reorganization and functional recovery Translational Neuroscience 2011 2 1 33 42 10.2478/s13380-011-0006-5

[B30] Blackman JA Early intervention: a global perspective Infants and Young Children 2002 15 2 11 19 10.1097/00001163-200210000-00004

[B31] McCarton CM Wallace IF Bennett FC Preventive interventions with low birth weight premature infants: An evaluation of their success Semin Perinatol 1995 19 4 330 340 10.1016/S0146-0005(05)80049-7 8560300

[B32] Orton J Spittle A Doyle L Anderson P Boyd R Do early intervention programmes improve cognitive and motor outcomes for preterm infants after discharge? A systematic review Dev Med Child Neurol 2009 51 11 851 859 10.1111/j.1469-8749.2009.03414.x 19732117

[B33] Spittle AJ Ferretti C Anderson PJ Orton J Eeles A Bates L Boyd RN Inder TE Doyle LW Improving the outcome of infants born at <30 weeks' gestation - a randomized controlled trial of preventative care at home BMC Pediatrics 2009 9 73 86 10.1186/1471-2431-9-73 19954550 PMC2797495

[B34] Vanderveen JA Bassler D Robertson CMT Kirpalani H Early interventions involving parents to improve neurodevelopmental outcomes of premature infants: a meta-analysis J Perinatol 2009 29 343 351 10.1038/jp.2008.229 19148113

[B35] Spittle A Orton J Doyle LW Boyd R Early developmental intervention programs post hospital discharge to prevent motor and cognitive impairments in preterm infants Cochrane Database of Systematic Reviews 2007 2 10.1002/14651858.CD005495.pub2 17443595

[B36] Pridham KA Lutz KF Anderson LS Riesch SK Becker PT Furthering the understanding of parent-child relationships: A nursing scholarship review series. Part 3: Interaction and the parent-child relationship - assessment and intervention studies Journal for Specialists in Pediatric Nursing 2009 15 1 33 61 20074112 10.1111/j.1744-6155.2009.00216.x PMC2835364

[B37] Lutz KF Anderson LS Riesch SK Pridham KA Becker PT Furthering the understanding of parent-child relationships: A nursing scholarship review series. Part 2: Grasping the early parenting experience - the insider view Journal for Specialists in Pediatric Nursing 2009 14 4 262 283 10.1111/j.1744-6155.2009.00209.x 19796326 PMC2835347

[B38] Melnyk BM Alpert-Gillis L Feinstein NF Fairbanks E Schultz-Czarniak J Hust D Sherman L LeMoine C Moldenhauer Z Small L Improving cognitive development of low-birth-weight premature infants with the COPE Program: A pilot study of the Benefit of early NICU intervention with mothers Res Nurs Health 2001 24 373 389 10.1002/nur.1038 11746067

[B39] Melnyk BM Feinstein NF Alpert-Gillis L Fairbanks E Crean HF Sinkin RA Stone PW Small L Tu X Gross SJ Reducing premature infants' length of stay and improving parents' mental health outcomes with the Creating Opportunities for Parent Empowerment (COPE) neonatal intensive care unit program: A randomized, controlled trial Pediatrics 2006 118 5 e1414 e1427 10.1542/peds.2005-2580 17043133

[B40] IHDP - The Infant Health and Development Program Enhancing the outcomes of low-birth-weight, premature infants: A multisite, randomized trial Journal of the American Medical Association 1990 263 22 3035 3042 2188023 10.1001/jama.1990.03440220059030

[B41] Als H Gilkerson L The role of relationship-based developmentally supportive newborn intensive care in strengthening outcome of preterm infants Semin Perinatol 1997 21 3 178 189 10.1016/S0146-0005(97)80062-6 9205974

[B42] ProQuest RefWorks 2008

[B43] The Cochrane Collaboration Review Manager (RevMan) 2011 5.1 Copenhagen: The Nordic Cochrane Centre

[B44] Higgins J Green S Cochrane handbook for systematic reviews of interventions 2011 5.1.0 The Cochrane Collaboration

[B45] Sterne JAC Egger M Funnel plots for detecting bias in meta-analysis: Guidelines on choice of axis J Clin Epidemiol 2001 54 10 1046 1055 10.1016/S0895-4356(01)00377-8 11576817

[B46] Sajaniemi N Makela J Salokorpi T von Wendt L Hamalainen T Hakamies-Blomqvist L Cognitive performance and attachment patterns at four years of age in extremely low birth weight infants after early intervention European Child and Adolescent Psychology 2001 10 2 122 129 10.1007/s007870170035 11469284

[B47] Bagner DM Sheinkopf SJ Vohr BR Lester BM Parenting intervention for externalizing behavior problems in children born premature: An initial examination J Dev Behav Pediatr 2010 31 3 209 216 10.1097/DBP.0b013e3181d5a294 20375736 PMC2866142

[B48] Ravn IH Smith L Lindemann R Smeby NA Kyno NM Bunch EH Sandvik L Effect of early intervention on social interaction between mothers and preterm infants at 12 months of age: A randomized controlled trial Infant Behavior & Development 2011 34 2 215 225 10.1016/j.infbeh.2010.11.004 21371754

[B49] Newnham CA Milgrom J Skouteris H Effectiveness of a modified mother–infant transaction program on outcomes for preterm infants from 3 to 24 months of age Infant Behavior & Development 2009 32 1 17 26 10.1016/j.infbeh.2008.09.004 19026450

[B50] Spittle AJ Anderson PJ Lee KJ Ferretti C Eeles A Orton J Boyd RN Inder T Doyle LW Preventive care at home for very preterm infants improves infant and caregiver outcomes at 2 years Pediatrics 2010 126 1 e171 e178 10.1542/peds.2009-3137 20547650

[B51] APIP (Avon Premature Infant Project) Randomised trial of parental support for families with very preterm children Archives of Disease in Childhood Fetal and Neonatal Edition 1998 79 F4 F11 10.1136/fn.79.1.F4 9797618 PMC1720827

[B52] Glazebrook C Marlow N Israel C Croudace T Johnson S White I Whitelaw A Randomised trial of a parenting intervention during neonatal intensive care Arch Dis Child 2007 92 F438 F443 10.1136/adc.2006.103135 PMC2675386 17301114

[B53] Brisch KH Bechinger D Betzler S Heinemann H Early preventive attachment-oriented psychotherapeutic intervention program with parents of a very low birthweight premature infant: Results of attachment and neurological development Attachment & Human Development 2003 5 2 120 135 10.1080/1461673031000108504 12791563

[B54] Gianni ML Picciolini O Ravasi M Gardon L Vegni C Fumagalli M Mosca F The effects of an early developmental mother—child intervention program on neurodevelopment outcome in very low birth weight infants: A pilot study Early Hum Dev 2006 82 10 691 695 10.1016/j.earlhumdev.2006.01.011 16530990

[B55] Ohgi S Fukuda M Akiyama T Gima H Effect of an early intervention programme on low birthweight infants with cerebral injuries Journal of Paediatric and Child Health 2004 40 12 689 695 10.1111/j.1440-1754.2004.00512.x 15569286

[B56] Meijssen D Wolf MJ Koldewijn K Houtzager BA van Wassenaer A Tronick E Kok J van Baar A The effect of the Infant Behavioral Assessment and Intervention Program on mother–infant interaction after very preterm birth The Journal of Child Psychology and Psychiatry 2010 51 11 1287 1295 10.1111/j.1469-7610.2010.02237.x 20345840

[B57] Kang R Barnard K Hammond M Oshio S Spencer C Thibodeaux B Williams J Preterm infant follow-up project: A multi-site field experiment of hospital and home intervention programs for mothers and preterm infants Public Health Nurs 1995 12 3 171 180 10.1111/j.1525-1446.1995.tb00006.x 7596966

[B58] Neu M Robinson J Maternal holding of preterm infants during the early weeks after birth and dyad interaction at six months J Obstet Gynecol Neonatal Nurs 2010 39 4 401 414 10.1111/j.1552-6909.2010.01152.x PMC2935695 20629927

[B59] O'Connell MA Maternal sensitivity in a high-risk, African American preterm sample: An ecological approach to predictors and effects of interventions 2007 University of Maryland, Psychology

[B60] Saylor CF Casto G Huntington L Predictors of developmental outcomes for medically fragile early intervention participants J Pediatr Psychol 1996 21 869 887 10.1093/jpepsy/21.6.869 8990730

[B61] Teti DM Black MM Viscardi R Glass P O'Connell MA Baker L Cusson R Reiner Hess C Intervention with African American premature infants: Four-month results of an early intervention program Journal of Early Intervention 2009 31 2 146 166 10.1177/1053815109331864

[B62] Koldewijn K Wolf MJ van Wassenaer A Meijssen D van Sonderen L Van Baar A Beelen A Nollet F Kok J The infant behavioral assessment and intervention program for very low birth weight infants at 6 months corrected age The Journal of Pediatrics 2009 154 1 33 38 10.1016/j.jpeds.2008.07.039 18783797

[B63] McCormick MC Brooks-Gunn J Buka SL Goldman J Yu J Salganik M Scott DT Bennett FC Kay LL Bernbaum JC Early intervention in low birth weight premature infants: Results at 18 years of age for the infant health and development program Pediatrics 2006 117 3 771 780 10.1542/peds.2005-1316 16510657

[B64] Milgrom J Newnham CA Anderson PJ Doyle LW Gemmill AW Lee K Hunt RW Bear M Inder T Early sensitivity training for parents of preterm infants: Impact on the developing brain Pediatr Res 2010 67 3 330 335 10.1203/PDR.0b013e3181cb8e2f 19952869

[B65] Meijssen DE Wolf MJ Koldewijn K van Wassenaer AG Kok JH van Baar AL Parenting stress in mothers after very preterm birth and the effect of the Infant Behavioral Assessment and Intervention Program Child Care Health Dev 2010 37 2 195 202 20645992 10.1111/j.1365-2214.2010.01119.x

[B66] Meijssen D Wolf MJ van Bakel H Koldewijn K Kok J van Baar A Maternal attachment representations after very preterm birth and the effect of early intervention Infant Behavior & Development 2011 34 1 72 80 10.1016/j.infbeh.2010.09.009 21067812

[B67] IHDP (The Infant Health and Development Program) Enhancing the outcomes of low-birth-weight, premature infants: A multisite, randomized trial Journal of the American Medical Association 1990 263 22 3035 3042 2188023 10.1001/jama.1990.03440220059030

[B68] Egeland B Weinfield NS Bosquet M Cheng VK Osofsky JD, Fitzgerald HE Remembering, repeating, and working through: Lessons from attachment-based interventions Handbook of infant mental health Vol 4: Infant mental health in groups at high risk 2000 New York: Wiley 35 89

[B69] Bakermans-Kranenburg MJ van Ijzendoorn MH Juffer F Less is more: Meta-analyses of sensitivity and attachment interventions in early childhood Psychol Bull 2003 129 2 195 215 12696839 10.1037/0033-2909.129.2.195

[B70] Abidin RR Parenting Stress Index: Professional manual 1995 3 Odessa, FL: Psychological Assessment Resources

[B71] Miles MS Funk SG Carlson J Parental stressor scale: neonatal intensive care unit Nurs Res 1993 42 3 148 152 8506163

[B72] Nordhov SM Ronning JA Dahl LB Ulvund SE Tunby J Kaaresen PI Early intervention improves cognitive outcomes for preterm infants: Randomized controlled trial Pediatrics 2010 126 5 e1088 e1094 10.1542/peds.2010-0778 20937650

[B73] Spielberger CD Gorsuch RL Lushene RE Test manual for the state-trait anxiety inventory 1970 Palo Alto, CA: Consulting Psychologists Press

[B74] Zigmond AS Snaith RP The hospital anxiety and depression scale Acta Psychiatr Scand 1983 67 6 361 370 10.1111/j.1600-0447.1983.tb09716.x 6880820

[B75] Sumner G Speitz A NCAST Caregiver/Parent-Child Interaction Teaching Manual 1994 Seattle, WA: NCAST Publications, University of Washington, School of Nursing

[B76] Cox MJ Crnic K Qualitative ratings for parent-child interaction at 3-12 months of age 2002 Chapel Hill: University of North Carolina

[B77] Ballantyne M Benzies KM Trute B [Working title] Predictors of depressive symptoms among immigrant and Canadian born mothers of preterm infants at neonatal intensive care discharge: a cross sectional survey In progress 10.1186/1471-2393-13-S1-S11 PMC3561187 23445606

[B78] Miles MS Funk SG Kasper MA The stress response of mothers and fathers of preterm infants Res Nurs Health 1992 15 4 261 269 10.1002/nur.4770150405 1496151

[B79] Harrison MJ Magill-Evans J Mother and father interactions over the first year with term and preterm infants Res Nurs Health 1996 19 6 451 459 10.1002/(SICI)1098-240X(199612)19:6<451::AID-NUR1>3.0.CO;2-N 8948399

[B80] Nosarti C Giouroukou E Micali N Rifkin L Morris RG Murray RM Impaired executive functioning in young adults born very preterm J Int Neuropsychol Soc 2007 13 1 11 17521479 10.1017/S1355617707070725

[B81] Olafsen KS Ronning JA Kaaresen PI Ulvund SE Handegard BH Dahl LB Joint attention in term and preterm infants at 12 months corrected age: The significance of gender and intervention based on a randomized controlled trial Infant Behavior & Development 2006 29 4 554 563 10.1016/j.infbeh.2006.07.004 17138308

