# Supplementary material for: Key components of early intervention programs for preterm infants and their parents: a systematic review and meta-analysis
Source: BMC Pregnancy Childbirth. 2013 Jan 31;13(Suppl 1):S10. doi: 10.1186/1471-2393-13-S1-S10 (PMC3561170; doi:10.1186/1471-2393-13-S1-S10)
Supplement: Additional File — Supplementary Table - Final search strategy [file 1471-2393-13-S1-S10-S1.docx]

Supplementary Table. Final search strategy

MEDLINE Search

Database: Ovid MEDLINE(R) <1950 to November Week 3 2010> Search Strategy:

1 Mother-child relations/ or Maternal behavior/ or Parent-child relations/ or Paternal behavior/ or Parenting/

2 exp Parents/ (

3 exp Mothers/

4 exp Fathers/)

5 (Early intervention$ or program$ or parental sensitivity or parental responsiveness or home visit$ or education or behavior modification or treatment or therap$ or outcome assessment or anticipatory guidance or counseling or developmental care or infant stimulation or intervention).mp. [mp=title, original title, abstract, name of substance word, subject heading word, unique identifier]

6 late preterm.mp.

7 near term.mp.

8 exp Gestational Age/

9 exp Infant, Premature/

10 exp Premature Birth/

11 6 or 7 or 8 or 9 or 10

12 1 or 2 or 3 or 4

13 5 and 11 and 12

14 limit 13 to (English language and humans and yr="1990 -Current")
